# Effectors of plant-colonizing fungi and beyond

**DOI:** 10.1371/journal.ppat.1006992

**Published:** 2018-06-07

**Authors:** Simon Uhse, Armin Djamei

**Affiliations:** Gregor Mendel Institute (GMI), Austrian Academy of Sciences, Vienna BioCenter (VBC), Vienna, Austria; THE SAINSBURY LABORATORY, UNITED KINGDOM

Plant–microbe interactions have evolved over hundreds of millions of years, generating a diversity of interactions covering a broad continuum from pathogenic to mutualistic coexistence. Although these different lifestyles have different needs, they all bear in common the use of secreted molecules, termed “effectors”, that enable microbes to interact with their hosts and to influence the outcome of the interaction. Effectors are not distinguished by sharing similar chemical properties but are instead defined by their function within the biological context of an interaction. To understand effectors, one needs to understand the coevolutionary forces that shape them. The host defense system is a major selection force that eradicates pathogens with a nonadapted effector repertoire. Reciprocally, host plants only survive the evolutionary race if they have been selected to recognize and defend against invading pathogens. This ongoing coevolution creates complex interdependencies between the effector repertoire of microbes, their effectome, and the host susceptibility machinery and defense system of their host plants. This review will summarize recent advances made in the field of effector studies in filamentous plant-colonizing microbes.

## Effector gene expression—Being in the right place at the right time

Each produced effector can be considered as an investment that needs to pay off by giving a selective advantage to the invader, at least from time to time across generations, to be kept in the population. As many effectors are tools that redirect host metabolism and development, their dosage and timing should be controlled to achieve an optimal, balanced result, especially in the case of biotrophs, which need to retain the viability of their host. Evidence for the tight control of effector synthesis and their place and mode of secretion has been provided from various filamentous pathogens [1–4]. Lifestyle switches, e.g., from biotrophic to necrotrophic, or host switches require profound changes in the applied effector cocktail [5]. The same is true when changing environments within the host, e.g., by moving between organs, as exemplified for the biotrophic maize pathogen *Ustilago maydis* [6]. Growing evidence supports the view that adapting the composition of produced effectors to external cues and developmental requirements is a general feature of interspecies interactions. Infection-phase–specific expression of putative effectors has been demonstrated by transcriptomic time-course experiments, among others, in the obligate biotrophic poplar leaf rust *Melampsora larici-populina* [7]; the hemibiotrophic fungus *Colletotrichum higginsianum*, which causes anthracnose during *Arabidopsis thaliana* infection [8]; the obligate biotrophic barley fungus *Blumeria graminis* [9]; the root mutualistic fungus *Serendipita indica* (former *Piriformospora indica*) [5]; and the maize-infecting biotroph *U*. *maydis* [10]. Adaptation of effector secretion and/or expression may even be cell-type-specific, although this hypothesis lacks experimental support, likely because of technical challenges. An emerging concept is that adaptation of effector expression is not limited to developmental programs of the pathogen or infection strategies in different hosts or plant organs but also occurs when the host plant is challenged by abiotic stresses. Transcriptomic studies on rice under mild drought stress showed that the hemibiotrophic fungus *Magnaporthe oryzae* transcriptionally downregulates the majority of its putative effectors despite being more successful in colonizing the stressed plants [11]. All these examples of adapted effector expression imply that specific environmental signals must be perceived during colonization by the invading microbes. On the pathogen side, very little is known about what these external signals are and how they are perceived, especially after infection [12, 13]. As misregulation of effectors has been shown to reduce pathogenicity in various pathogens, manipulating effector expression via these external cues could be an elegant way to interfere with pathogen infections [4, 14]. Studying the underlying regulatory networks controlling effector expression is an important future research direction.

## Enigmatic effector translocation and place of action

A common hallmark of effectors is that they are, in one way or the other, secreted. Their place of action is therefore either in the interphase between the microbe and the host cell (apoplastic effectors) or inside the host cell (translocated/symplastic effectors). The term “symplastic effector” embodies the idea that translocated effectors might not be restricted to a single cell and includes all possible places of action within plant cells. Similar to the spreading of effectors within the symplast, effectors might diffuse within the apoplast and therefore act on several cells. Within these two compartments, further subcompartments can be delimited. Within the apoplast, effectors have been identified that bind fungal cell wall components, potentially to protect their degradation or recognition by plant pattern-recognition receptors [15, 16]. Other effectors act in the biotrophic interphase, e.g., as inhibitors of apoplastic proteases or to bind pathogen-associated molecular patterns (PAMPs) to reduce recognition [17, 18].

We are not aware of any effector being identified with targets associated with the host plasma membrane from the apoplastic side, and only a few have been identified acting from the cytosolic side at the membrane, likely because of technical limitations in identifying these interactions [19–21].

Type III secretion signals from bacteria and RXLR-dEER or LXLFLAK motifs from oomycetes are predicted to be translocation signals (although in case of RXLR-dEER, its role in uptake is under debate [22]), which make the prediction of symplastic effectors possible in these systems [23, 24]. For fungi, RXLR-like signals leading to translocation of fungal effectors have been controversially discussed but have not been confirmed [25, 26]. Experimental evidence for translocation has been generated either directly by fusing fluorescent proteins to effectors [27] or through immunoelectron-microscopy approaches [28, 29] or are inferred by cytosolic resistance gene (R-gene)–based recognition of avirulence (Avr) effectors [30]. Experimental results for the rust symplastic effector AvrM indicate a host-cell autonomous translocation [29, 31], which implies that AvrM harbors intrinsic biochemical properties mediating its translocation. In contrast to this, the effector Avr2 of *Fusarium oxysporum* does not show such properties but instead requires a pathogen-derived trigger for translocation [32]. The differences observed between pathosystems make it likely that the mechanisms of translocation into the host cell might differ between fungal species and potentially even between different symplastic effectors within a species [27–29, 33]. After translocation into the host cell, symplastic effectors might target specific host compartments. Transgenic production of effector proteins without signal peptides in plant cells have indicated specific localization for effectors in the nucleus, nucleoli, chloroplasts, mitochondria, and discrete cellular bodies [34, 35].

## Effector functions—Avoid the alarm, activate what serves, and inhibit what harms

The functions that need to be covered by an effectome reflect the challenges presented by the host immune machinery and mirror the specific needs of the pathogen and its lifestyle. While effectors of biotrophs often function in suppression of host immunity, the necrotrophic fungus *Cochliobolus victoriae* targets a defense-associated thioredoxin TRX-h5 guarded by the NB-LRR protein LOV1 via the toxin effector victorin. The LRR recognition leads to host defense responses, conferring disease susceptibility to the necrotroph [36].

**Fig 1:**
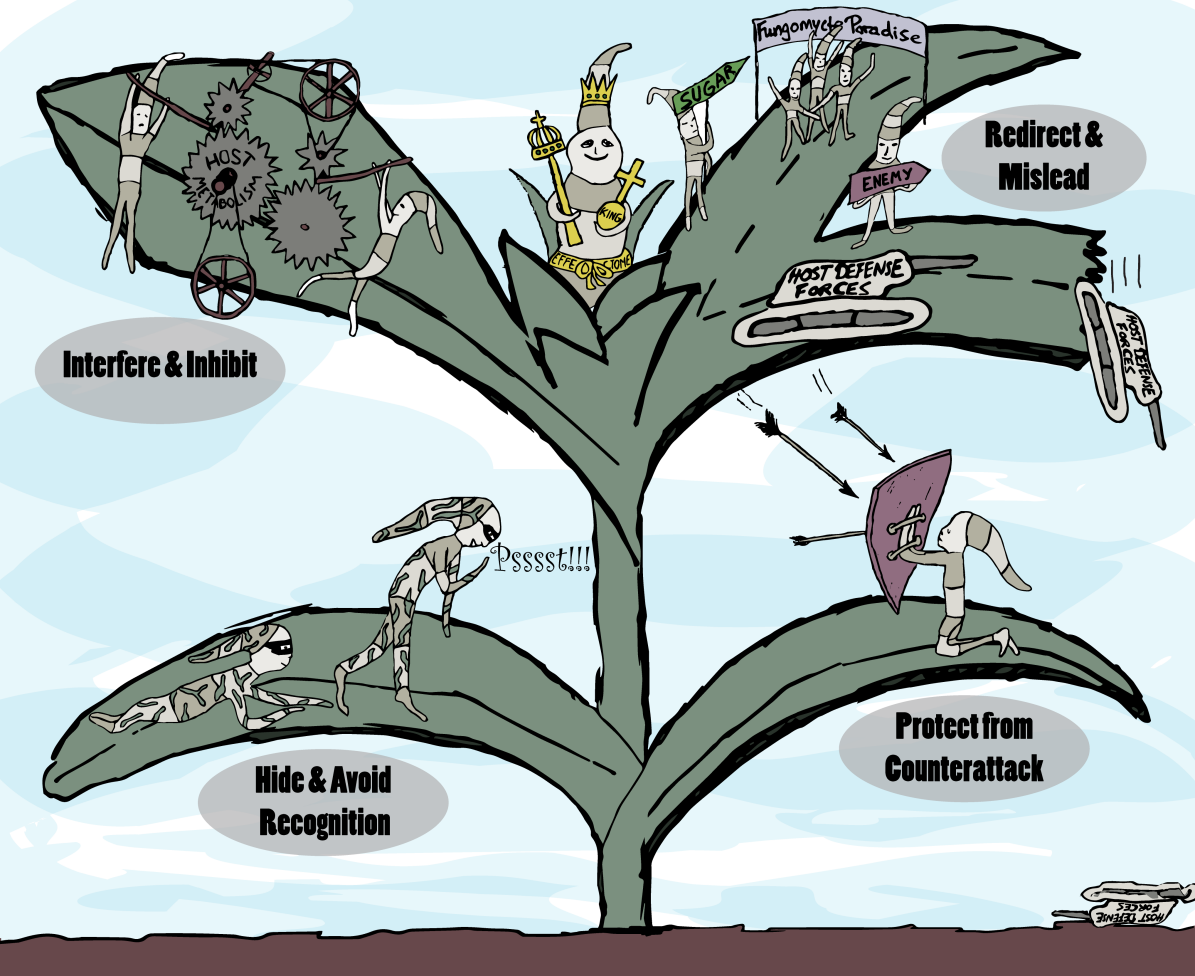
Strategies for successful host invasion. Plant-colonizing microbes employ effectors fulfilling various functions during the host invasion, which are visualized symbolically in this cartoon. Different modes of action (self-binding and self-modifying, activating or inhibiting activities) of effectors described in the text may be applied to serve the listed strategies (text on grey oval background).

Looking at so-far-identified effector functions, one can identify different modes of action serving the strategies for successful host invasion illustrated in Fig 1.

### The self-binder and self-modifier

Effectors with a defensive mode of action either sequester potential microbe-associated molecular patterns (MAMPs) or modify their cell walls upon penetration to minimize recognition. Examples include the chitin-oligomer-chelating LysM effectors Ecp6 of *Cladosporium fulvum* or the Slp1 LysM effector of *M*. *oryzae* [18, 37]. Another effector passively protects from antimicrobial counter attack [16, 38].

### The inhibitor

Many effectors have classic inhibitory activities, e.g., against immune-related proteases, glucanases, or peroxidases, but also against intracellular signaling components to interfere with defense-related signaling processes [39–42]. Inhibition of the Jasmonic-acid–triggered degradation of PtJAZ6 by the MiSSP7 *Laccaria bicolor* effector is an example of signaling suppression by a mutualistic fungus [43].

### The activator

Only a few effectors have been identified that clearly fall into the activator category, probably as evolution of inhibitory activity is more likely. The NUDIX hydrolase effector Avr3b of *Phytophthora sojae* and the deregulated, secreted chorismate mutase Cmu1 of *U*. *maydis* are examples [28, 44]. Some activating effectors function by interfering with the deactivation or degradation of their interacting host protein, thereby acting positively, although they are basically an inhibitor type of effector. One example is the *U*. *maydis* effector Tin2, which stabilizes the maize kinase TKK1 by inhibiting its degradation [33].

Most effector functions are usually inferred via the host interaction partners, as many effectors show low conservation on the sequence level because of high selection pressure to evade host recognition. One conceptional restriction is that effectors might interact with host molecules either to target and manipulate them or to use them as part of the host cellular machinery to reach their final destination. For example, an effector with a nuclear localization signal might interact with Importin α to enter the host nucleus, but its ultimate target might be the inhibition of a specific host transcription factor. Some effectors have a broader target spectrum, as exemplified by EPIC2B, a cystatin-domain-containing, protease-inhibiting effector from *Phytophtora infestans* [45]. Other effectors show a high degree of specificity even when they target members of expanded protein families, as is the case for the *M*. *oryzae* effector Avr-Pii, which targets specific vesicle-tethering Exo70 subunits involved in host immune responses, or the *P*. *infestans* effector PexRD54, which targets a specific autophagy-modulating ubiquitin-like ATG8 family member [46, 47].

Large-scale effector/host ORFeome interaction screens demonstrated that effector targets are usually well-connected cellular hubs [48, 49]. Furthermore, these and other studies revealed that effectors often converge on the same host targets [50]. This goes hand in hand with independent observations that many effector deletion strains do not show any observable virulence defect, potentially a reflection of functional redundancy [51]. Functional redundancy likely provides robustness to host-colonization success and could be considered a sign that the target is of specific importance for a successful interaction. This is supported by a correlation between converging effector-target–deletion plants often showing altered immune-response phenotypes [49].

The decoy-domain fusions found in many nucleotide binding domain and leucine-rich repeat receptor (NLR) proteins might represent effector-target mimics. This, among others, has been experimentally validated for the WRKY domain containing NLR RRS1-R [52]. Therefore, sensor domains fused to NLRs might serve as an informative way to preselect common effector targets [53]. While effectors also target directly defense components, they more commonly target defense modulators, e.g., by exploiting antagonistic hormone pathways that promote both growth and development, thereby inhibiting immunity [48]. This could be a coevolutionary consequence of the host immune system being less able to detect manipulation of modulators that are involved in various processes beyond immunity.

## Outlook

Within the context of the host metabolism, effectors act as alien molecules, overrunning feedback control systems that usually maintain homeostasis [33]. For this reason, they are valuable dominant acting molecular tools. Effectors teach us not only about the molecular defense machinery of the host but often disclose the wiring between immunity, growth, and developmental host pathways. Like a molecular language, effectors coevolve with the host population the invader needs to communicate with. Our understanding of this language is still in the early stages, and thousands of effectomes await to be understood. However, being able to translate this language will likely reward us with immense payback both in strategies for preventing pathogen infections and tools for understanding plant biology.

## References

[1] Bielska E, Higuchi Y, Schuster M, Steinberg N, Kilaru S, Talbot NJ, et al Long-distance endosome trafficking drives fungal effector production during plant infection. Nat Commun. 2014;5:5097 doi: 10.1038/ncomms6097 . 25283249 10.1038/ncomms6097 PMC4205857

[2] Giraldo MC, Dagdas YF, Gupta YK, Mentlak TA, Yi M, Martinez-Rocha AL, et al Two distinct secretion systems facilitate tissue invasion by the rice blast fungus Magnaporthe oryzae. Nat Commun. 2013;4 doi: 10.1038/ncomms2996 23774898 10.1038/ncomms2996 PMC3709508

[3] Wang S, Boevink PC, Welsh L, Zhang R, Whisson SC, Birch PRJ. Delivery of cytoplasmic and apoplastic effectors from Phytophthora infestans haustoria by distinct secretion pathways. New Phytol. 2017;216(1):205–15. doi: 10.1111/nph.14696 . 28758684 10.1111/nph.14696 PMC5601276

[4] Tollot M, Assmann D, Becker C, Altmuller J, Dutheil JY, Wegner CE, et al The WOPR Protein Ros1 Is a Master Regulator of Sporogenesis and Late Effector Gene Expression in the Maize Pathogen Ustilago maydis. PLoS Pathog. 2016;12(6). doi: 10.1371/journal.ppat.1005697 27332891 10.1371/journal.ppat.1005697 PMC4917244

[5] Lahrmann U, Ding Y, Banhara A, Rath M, Hajirezaei MR, Dohlemann S, et al Host-related metabolic cues affect colonization strategies of a root endophyte. Proc Natl Acad Sci USA. 2013;110(34):13965–70. doi: 10.1073/pnas.1301653110 . 23918389 10.1073/pnas.1301653110 PMC3752250

[6] Skibbe DS, Doehlemann G, Fernandes J, Walbot V. Maize tumors caused by Ustilago maydis require organ-specific genes in host and pathogen. Science. 2010;328(5974):89–92. Epub 2010/04/03. doi: 10.1126/science.1185775 . 20360107 10.1126/science.1185775

[7] Duplessis S, Hacquard S, Delaruelle C, Tisserant E, Frey P, Martin F, et al Melampsora larici-populina Transcript Profiling During Germination and Timecourse Infection of Poplar Leaves Reveals Dynamic Expression Patterns Associated with Virulence and Biotrophy. Mol Plant Microbe In. 2011;24(7):808–18. doi: 10.1094/Mpmi-01-11-0006 21644839 10.1094/MPMI-01-11-0006

[8] O'Connell RJ, Thon MR, Hacquard S, Amyotte SG, Kleemann J, Torres MF, et al Lifestyle transitions in plant pathogenic Colletotrichum fungi deciphered by genome and transcriptome analyses. Nat Genet. 2012;44(9):1060–5. doi: 10.1038/ng.2372 . 22885923 10.1038/ng.2372 PMC9754331

[9] Hacquard S, Kracher B, Maekawa T, Vernaldi S, Schulze-Lefert P, Ver Loren van Themaat E. Mosaic genome structure of the barley powdery mildew pathogen and conservation of transcriptional programs in divergent hosts. Proc Natl Acad Sci USA. 2013;110(24):E2219–28. doi: 10.1073/pnas.1306807110 . 23696672 10.1073/pnas.1306807110 PMC3683789

[10] Lanver D, Muller AN, Happel P, Schweizer G, Haas FB, Franitza M, et al The biotrophic development of Ustilago maydis studied by RNAseq analysis. Plant Cell. 2018 doi: 10.1105/tpc.17.00764 . 29371439 10.1105/tpc.17.00764 PMC5868686

[11] Bidzinski P, Ballini E, Ducasse A, Michel C, Zuluaga P, Genga A, et al Transcriptional Basis of Drought-Induced Susceptibility to the Rice Blast Fungus Magnaporthe oryzae. Front Plant Sci. 2016;7:1558 doi: 10.3389/fpls.2016.01558 . 27833621 10.3389/fpls.2016.01558 PMC5081564

[12] Mendoza-Mendoza A, Berndt P, Djamei A, Weise C, Linne U, Marahiel M, et al Physical-chemical plant-derived signals induce differentiation in Ustilago maydis. Mol Microbiol. 2009;71(4):895–911. Epub 2009/01/28. doi: 10.1111/j.1365-2958.2008.06567.x . 19170880 10.1111/j.1365-2958.2008.06567.x

[13] Lanver D, Mendoza-Mendoza A, Brachmann A, Kahmann R. Sho1 and Msb2-related proteins regulate appressorium development in the smut fungus Ustilago maydis. Plant Cell. 2010;22(6):2085–101. Epub 2010/07/01. doi: 10.1105/tpc.109.073734 . 20587773 10.1105/tpc.109.073734 PMC2910971

[14] Michielse CB, van Wijk R, Reijnen L, Manders EMM, Boas S, Olivain C, et al The Nuclear Protein Sge1 of Fusarium oxysporum Is Required for Parasitic Growth. PLoS Pathog. 2009;5(10). doi: 10.1371/journal.ppat.1000637 19851506 10.1371/journal.ppat.1000637 PMC2762075

[15] Wawra S, Fesel P, Widmer H, Timm M, Seibel J, Leson L, et al The fungal-specific beta-glucan-binding lectin FGB1 alters cell-wall composition and suppresses glucan-triggered immunity in plants. Nat Commun. 2016;7 doi: 10.1038/ncomms13188 27786272 10.1038/ncomms13188 PMC5095285

[16] van den Burg HA, Harrison SJ, Joosten MHAJ, Vervoort J, de Wit PJGM. Cladosporium fulvum Avr4 protects fungal cell walls against hydrolysis by plant chitinases accumulating during infection. Mol Plant Microbe In. 2006;19(12):1420–30. doi: 10.1094/Mpmi-19-1420 17153926 10.1094/MPMI-19-1420

[17] Mueller AN, Ziemann S, Treitschke S, Assmann D, Doehlemann G. Compatibility in the Ustilago maydis-Maize Interaction Requires Inhibition of Host Cysteine Proteases by the Fungal Effector Pit2. PLoS Pathog. 2013;9(2). doi: 10.1371/journal.ppat.1003177 23459172 10.1371/journal.ppat.1003177 PMC3573112

[18] de Jonge R, van Esse HP, Kombrink A, Shinya T, Desaki Y, Bours R, et al Conserved Fungal LysM Effector Ecp6 Prevents Chitin-Triggered Immunity in Plants. Science. 2010;329(5994):953–5. doi: 10.1126/science.1190859 . 20724636 10.1126/science.1190859

[19] Wu J, van der Burgh AM, Bi G, Zhang L, Alfano JR, Martin GB, et al The Bacterial Effector AvrPto Targets the Regulatory Coreceptor SOBIR1 and Suppresses Defense Signaling Mediated by the Receptor-Like Protein Cf-4. Mol Plant Microbe Interact. 2018;31(1):75–85. doi: 10.1094/MPMI-08-17-0203-FI . 28876174 10.1094/MPMI-08-17-0203-FI

[20] Lu D, He P, Shan L. Bacterial effectors target BAK1-associated receptor complexes: One stone two birds. Commun Integr Biol. 2010;3(2):80–3. . 20585495 10.4161/cib.3.2.10301 PMC2889959

[21] Cao L, Blekemolen MC, Tintor N, Cornelissen BJC, Takken FLW. The Fusarium oxysporum Avr2-Six5 Effector Pair Alters Plasmodesmatal Exclusion Selectivity Facilitating Cell-to-Cell Movement of Avr2. Mol Plant. 2018;11(5):691–705. doi: 10.1016/j.molp.2018.02.011 . 29481865 10.1016/j.molp.2018.02.011

[22] Wawra S, Trusch F, Matena A, Apostolakis K, Linne U, Zhukov I, et al The RxLR Motif of the Host Targeting Effector AVR3a of Phytophthora infestans Is Cleaved before Secretion. Plant Cell. 2017;29(6):1184–95. doi: 10.1105/tpc.16.00552 28522546 10.1105/tpc.16.00552 PMC5502441

[23] Birch PR, Rehmany AP, Pritchard L, Kamoun S, Beynon JL. Trafficking arms: oomycete effectors enter host plant cells. Trends Microbiol. 2006;14(1):8–11. Epub 2005/12/17. doi: 10.1016/j.tim.2005.11.007 . 16356717 10.1016/j.tim.2005.11.007

[24] Cornelis GR, Van Gijsegem F. Assembly and function of type III secretory systems. Annu Rev Microbiol. 2000;54:735–74. doi: 10.1146/annurev.micro.54.1.735 . 11018143 10.1146/annurev.micro.54.1.735

[25] Kale SD, Gu BA, Capelluto DGS, Dou DL, Feldman E, Rumore A, et al External Lipid PI3P Mediates Entry of Eukaryotic Pathogen Effectors into Plant and Animal Host Cells. Cell. 2010;142(2):284–95. doi: 10.1016/j.cell.2010.06.008 20655469 10.1016/j.cell.2010.06.008

[26] Wawra S, Djamei A, Albert I, Nurnberger T, Kahmann R, van West P. In vitro translocation experiments with RxLR-reporter fusion proteins of Avr1b from Phytophthora sojae and AVR3a from Phytophthora infestans fail to demonstrate specific autonomous uptake in plant and animal cells. Mol Plant Microbe Interact. 2013;26(5):528–36. Epub 2013/04/04. doi: 10.1094/MPMI-08-12-0200-R . 23547905 10.1094/MPMI-08-12-0200-R

[27] Khang CH, Berruyer R, Giraldo MC, Kankanala P, Park SY, Czymmek K, et al Translocation of Magnaporthe oryzae Effectors into Rice Cells and Their Subsequent Cell-to-Cell Movement. Plant Cell. 2010;22(4):1388–403. doi: 10.1105/tpc.109.069666 20435900 10.1105/tpc.109.069666 PMC2879738

[28] Djamei A, Schipper K, Rabe F, Ghosh A, Vincon V, Kahnt J, et al Metabolic priming by a secreted fungal effector. Nature. 2011;478(7369):395–8. Epub 2011/10/07. doi: 10.1038/nature10454 . 21976020 10.1038/nature10454

[29] Rafiqi M, Gan PH, Ravensdale M, Lawrence GJ, Ellis JG, Jones DA, et al Internalization of flax rust avirulence proteins into flax and tobacco cells can occur in the absence of the pathogen. Plant Cell. 2010;22(6):2017–32. Epub 2010/06/08. doi: 10.1105/tpc.109.072983 . 20525849 10.1105/tpc.109.072983 PMC2910983

[30] Dodds PN, Lawrence GJ, Catanzariti AM, Teh T, Wang CI, Ayliffe MA, et al Direct protein interaction underlies gene-for-gene specificity and coevolution of the flax resistance genes and flax rust avirulence genes. Proc Natl Acad Sci USA. 2006;103(23):8888–93. doi: 10.1073/pnas.0602577103 . 16731621 10.1073/pnas.0602577103 PMC1482673

[31] Ve T, Williams SJ, Catanzariti AM, Rafiqi M, Rahman M, Ellis JG, et al Structures of the flax-rust effector AvrM reveal insights into the molecular basis of plant-cell entry and effector-triggered immunity. P Natl Acad Sci USA. 2013;110(43):17594–9. doi: 10.1073/pnas.1307614110 24101475 10.1073/pnas.1307614110 PMC3808616

[32] Di X, Gomila J, Ma L, van den Burg HA, Takken FL. Uptake of the Fusarium Effector Avr2 by Tomato Is Not a Cell Autonomous Event. Front Plant Sci. 2016;7:1915 doi: 10.3389/fpls.2016.01915 . 28066471 10.3389/fpls.2016.01915 PMC5175262

[33] Tanaka S, Brefort T, Neidig N, Djamei A, Kahnt J, Vermerris W, et al A secreted Ustilago maydis effector promotes virulence by targeting anthocyanin biosynthesis in maize. Elife. 2014;3: e01355 doi: 10.7554/eLife.01355 24473076 10.7554/eLife.01355 PMC3904489

[34] Petre B, Saunders DGO, Sklenar J, Lorrain C, Win J, Duplessis S, et al Candidate Effector Proteins of the Rust Pathogen Melampsora larici-populina Target Diverse Plant Cell Compartments. Mol Plant Microbe In. 2015;28(6):689–700. doi: 10.1094/Mpmi-01-15-0003-R 25650830 10.1094/MPMI-01-15-0003-R

[35] Sperschneider J, Catanzariti AM, DeBoer K, Petre B, Gardiner DM, Singh KB, et al LOCALIZER: subcellular localization prediction of both plant and effector proteins in the plant cell. Sci Rep-Uk. 2017;7 doi: 10.1038/srep44598 28300209 10.1038/srep44598 PMC5353544

[36] Lorang J, Kidarsa T, Bradford CS, Gilbert B, Curtis M, Tzeng SC, et al Tricking the Guard: Exploiting Plant Defense for Disease Susceptibility. Science. 2012;338(6107):659–62. doi: 10.1126/science.1226743 23087001 10.1126/science.1226743 PMC4125361

[37] Mentlak TA, Kombrink A, Shinya T, Ryder LS, Otomo I, Saitoh H, et al Effector-Mediated Suppression of Chitin-Triggered Immunity by Magnaporthe oryzae Is Necessary for Rice Blast Disease. Plant Cell. 2012;24(1):322–35. doi: 10.1105/tpc.111.092957 22267486 10.1105/tpc.111.092957 PMC3289562

[38] van Esse HP, Bolton MD, Stergiopoulos I, de Wit PJGM, Thomma BPHJ. The chitin-binding Cladosporium fulvum effector protein Avr4 is a virulence factor. Mol Plant Microbe In. 2007;20(9):1092–101. doi: 10.1094/Mpmi-20-9-1092 17849712 10.1094/MPMI-20-9-1092

[39] Shabab M, Shindo T, Gu C, Kaschani F, Pansuriya T, Chintha R, et al Fungal effector protein AVR2 targets diversifying defense-related Cys proteases of tomato. Plant Cell. 2008;20(4):1169–83. doi: 10.1105/tpc.107.056325 18451324 10.1105/tpc.107.056325 PMC2390736

[40] Rose JKC, Ham KS, Darvill AG, Albersheim P. Molecular cloning and characterization of glucanase inhibitor proteins: Coevolution of a counterdefense mechanism by plant pathogens. Plant Cell. 2002;14(6):1329–45. doi: 10.1105/tpc.002253 12084830 10.1105/tpc.002253 PMC150783

[41] Yamaguchi K, Yamada K, Ishikawa K, Yoshimura S, Hayashi N, Uchihashi K, et al A Receptor-like Cytoplasmic Kinase Targeted by a Plant Pathogen Effector Is Directly Phosphorylated by the Chitin Receptor and Mediates Rice Immunity. Cell Host & Microbe. 2013;13(3):347–57. doi: 10.1016/j.chom.2013.02.007 23498959 10.1016/j.chom.2013.02.007

[42] Hemetsberger C, Herrberger C, Zechmann B, Hillmer M, Doehlemann G. The Ustilago maydis effector Pep1 suppresses plant immunity by inhibition of host peroxidase activity. PLoS Pathog. 2012;8(5):e1002684 Epub 2012/05/17. doi: 10.1371/journal.ppat.1002684 . 22589719 10.1371/journal.ppat.1002684 PMC3349748

[43] Plett JM, Daguerre Y, Wittulsky S, Vayssieres A, Deveau A, Melton SJ, et al Effector MiSSP7 of the mutualistic fungus Laccaria bicolor stabilizes the Populus JAZ6 protein and represses jasmonic acid (JA) responsive genes. P Natl Acad Sci USA. 2014;111(22):8299–304. doi: 10.1073/pnas.1322671111 24847068 10.1073/pnas.1322671111 PMC4050555

[44] Dong SM, Yin WX, Kong GH, Yang XY, Qutob D, Chen QH, et al Phytophthora sojae Avirulence Effector Avr3b is a Secreted NADH and ADP-ribose Pyrophosphorylase that Modulates Plant Immunity. PLoS Pathog. 2011;7(11): e1002353 doi: 10.1371/journal.ppat.1002353 22102810 10.1371/journal.ppat.1002353 PMC3213090

[45] Tian MY, Win J, Song J, van der Hoorn R, van der Knaap E, Kamoun S. A Phytophthora infestans cystatin-like protein targets a novel tomato papain-like apoplastic protease. Plant Physiology. 2007;143(1):364–77. doi: 10.1104/pp.106.090050 17085509 10.1104/pp.106.090050 PMC1761951

[46] Dagdas YF, Belhaj K, Maqbool A, Chaparro-Garcia A, Pandey P, Petre B, et al An effector of the Irish potato famine pathogen antagonizes a host autophagy cargo receptor. Elife. 2016;5: e10856 doi: 10.7554/eLife.10856 26765567 10.7554/eLife.10856 PMC4775223

[47] Fujisaki K, Abe Y, Ito A, Saitoh H, Yoshida K, Kanzaki H, et al Rice Exo70 interacts with a fungal effector, AVR-Pii, and is required for AVR-Pii-triggered immunity. Plant Journal. 2015;83(5):875–87. doi: 10.1111/tpj.12934 26186703 10.1111/tpj.12934

[48] Mukhtar MS, Carvunis AR, Dreze M, Epple P, Steinbrenner J, Moore J, et al Independently evolved virulence effectors converge onto hubs in a plant immune system network. Science. 2011;333(6042):596–601. Epub 2011/07/30. doi: 10.1126/science.1203659 . 21798943 10.1126/science.1203659 PMC3170753

[49] Wessling R, Epple P, Altmann S, He YJ, Yang L, Henz SR, et al Convergent Targeting of a Common Host Protein-Network by Pathogen Effectors from Three Kingdoms of Life. Cell Host & Microbe. 2014;16(3):364–75. doi: 10.1016/j.chom.2014.08.004 25211078 10.1016/j.chom.2014.08.004 PMC4191710

[50] Song J, Win J, Tian MY, Schornack S, Kaschani F, Ilyas M, et al Apoplastic effectors secreted by two unrelated eukaryotic plant pathogens target the tomato defense protease Rcr3. P Natl Acad Sci USA. 2009;106(5):1654–9. doi: 10.1073/pnas.0809201106 19171904 10.1073/pnas.0809201106 PMC2635833

[51] Kamper J, Kahmann R, Bolker M, Ma LJ, Brefort T, Saville BJ, et al Insights from the genome of the biotrophic fungal plant pathogen Ustilago maydis. Nature. 2006;444(7115):97–101. Epub 2006/11/03. doi: 10.1038/nature05248 . 17080091 10.1038/nature05248

[52] Le Roux C, Huet G, Jauneau A, Camborde L, Tremousaygue D, Kraut A, et al A Receptor Pair with an Integrated Decoy Converts Pathogen Disabling of Transcription Factors to Immunity. Cell. 2015;161(5):1074–88. doi: 10.1016/j.cell.2015.04.025 26000483 10.1016/j.cell.2015.04.025

[53] Sarris PF, Cevik V, Dagdas G, Jones JDG, Krasileva KV. Comparative analysis of plant immune receptor architectures uncovers host proteins likely targeted by pathogens. BMC Biol. 2016;14 doi: 10.1186/s12915-016-0228-7 26891798 10.1186/s12915-016-0228-7 PMC4759884

